# Nanoscale Structural Plasticity of the Active Zone Matrix Modulates Presynaptic Function

**DOI:** 10.1016/j.celrep.2017.02.064

**Published:** 2017-03-14

**Authors:** Oleg O. Glebov, Rachel E. Jackson, Christian M. Winterflood, Dylan M. Owen, Ellen A. Barker, Patrick Doherty, Helge Ewers, Juan Burrone

**Affiliations:** 1 Wolfson Centre for Age-Related Diseases, Institute of Psychiatry, Psychology and Neuroscience, King’s College London, London SE1 1UL, UK; 2 Centre For Developmental Neurobiology, Institute of Psychiatry, Psychology and Neuroscience, King’s College London, London SE1 1UL, UK; 3 Randall Division of Molecular Biophysics, Faculty of Life Sciences and Medicine, King’s College London, London SE1 1UL, UK; 4 Department of Physics, Faculty of Natural and Mathematical Sciences, King’s College London, London WC2R 2LS, UK; 5 School of Biochemistry, University of Bristol, Bristol BS8 1TD, UK; 6 Institute of Chemistry and Biochemistry, Freie Universität Berlin, 14195 Berlin, Germany

**Keywords:** synaptic plasticity, super-resolution microscopy

## Abstract

The active zone (AZ) matrix of presynaptic terminals coordinates the recruitment of voltage-gated calcium channels (VGCCs) and synaptic vesicles to orchestrate neurotransmitter release. However, the spatial organization of the AZ and how it controls vesicle fusion remain poorly understood. Here, we employ super-resolution microscopy and ratiometric imaging to visualize the AZ structure on the nanoscale, revealing segregation between the AZ matrix, VGCCs, and putative release sites. Long-term blockade of neuronal activity leads to reversible AZ matrix unclustering and presynaptic actin depolymerization, allowing for enrichment of AZ machinery. Conversely, patterned optogenetic stimulation of postsynaptic neurons retrogradely enhanced AZ clustering. In individual synapses, AZ clustering was inversely correlated with local VGCC recruitment and vesicle cycling. Acute actin depolymerization led to rapid (5 min) nanoscale AZ matrix unclustering. We propose a model whereby neuronal activity modulates presynaptic function in a homeostatic manner by altering the clustering state of the AZ matrix.

## Highlights

• Dual-color STORM imaging of active zone (AZ) organization
• Neuronal activity bidirectionally regulates clustering of the AZ matrix
• Presynaptic machinery and function inversely correlate with AZ matrix clustering

## Graphical Abstract

**Figure:**
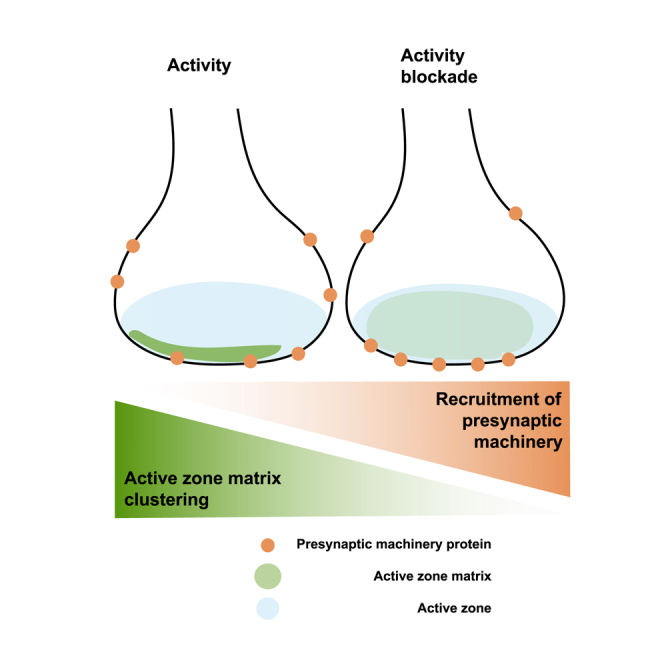

## Teaser

Glebov et al. find that the clustered presynaptic matrix restricts the recruitment of presynaptic machinery to the active zone. Neuronal activity bidirectionally controls clustering of the AZ matrix to control presynaptic function. These data suggest that AZ structure may locally control function through molecular crowding.

## Introduction

Synaptic transmission begins with the entry of calcium into the presynaptic terminal through voltage-gated calcium channels (VGCCs), followed by the fusion of neurotransmitter-filled synaptic vesicles (SV) with the presynaptic membrane. Both of these events occur at the active zone (AZ), a specialized site in presynaptic boutons that brings together VGCCs and synaptic vesicles within close proximity of each other and of release sites (Südhof, 2012).

Modulation of the AZ structure is thought to be an important regulatory site controlling the efficacy of synaptic transmission. The function of the AZ can be dynamically regulated in the context of homeostatic plasticity, resulting in increase in presynaptic Ca^2+^ influx (Zhao et al., 2011) and release probability (Murthy et al., 2001, Vitureira et al., 2011). Similarly, blockade of postsynaptic activity also results in structural changes in hippocampal boutons (Murthy et al., 2001) as well as numerous changes in the levels of AZ proteins in cortical synapses (Lazarevic et al., 2011), lending further support to the idea that the AZ structure is dynamic and controls presynaptic function. However, the connection between neuronal activity and the precise structure of the AZ has not been formally investigated.

Ultrastructurally, the AZ is formed of a dense scaffold containing hundreds of copies of dozens of different proteins. This tight arrangement is thought to result in a locally crowded molecular environment (Morales et al., 2000, Rothman et al., 2016, Wilhelm et al., 2014), that could lead to competition for space between AZ proteins. Due to its high molecular weight (410 kDa) and large estimated numbers (>400 molecules per AZ) (Wilhelm et al., 2014), the matrix protein Bassoon (Bsn) is estimated to account for a large proportion of the AZ matrix material. This is in contrast to the levels of other AZ proteins such as Rab3-interacting molecule (RIM) and VGCCs, whose numbers are estimated to be an order of magnitude lower (Holderith et al., 2012, Indriati et al., 2013, Nakamura et al., 2015, Wilhelm et al., 2014). As a result of its large bulk and high abundance, Bsn is a prime candidate for shaping the structure of the AZ matrix, with important implications for presynaptic function.

Theoretical simulations and electrophysiological and structural evidence suggest that the spatial organization of the AZ components may play an important role in coupling Ca^2+^ signaling and presynaptic release (Eggermann et al., 2011, Ermolyuk et al., 2013, Grauel et al., 2016, Holderith et al., 2012, Nakamura et al., 2015, Tang et al., 2016). Several key AZ proteins, including VGCCs, RIM, and Bsn, are non-randomly distributed in the AZ, raising the possibility that the structural arrangement of the AZ may be important for synapse function. In agreement with this, correlation between RIM levels and release probability (Holderith et al., 2012, Paul et al., 2015, Peled et al., 2014) and preferential occurrence of vesicle fusion in RIM-enriched domains (Tang et al., 2016) establishes RIM as a structural marker for release sites and of synapse strength. However, the relative spatial organization, as well as the dynamics, of the AZ components remains unknown.

Here, we hypothesized that the AZ architecture may be regulated by neuronal activity and that the structural plasticity of the AZ may be involved in controlling synapse function. To test this, we used high-resolution imaging to investigate the relationship between neuronal function and the nanoscale AZ structure in hippocampal synapses. Our results show that the AZ architecture features largely non-overlapping domains of Bsn-enriched matrix and either VGCCs or RIM-enriched areas located in close proximity to each other. We find that the clustering of Bsn is bidirectionally controlled by postsynaptic neuronal activity. Activity blockade results in Bsn unclustering and recruitment of multiple AZ proteins, while local Bsn clustering is negatively correlated with AZ protein enrichment and presynaptic function. Our data therefore suggest that clustering of the Bsn-enriched AZ matrix may act as a barrier to the recruitment of the presynaptic machinery, thus limiting synapse function. This restrictive modality constitutes a mechanism gating local presynaptic release, implicating molecular congestion in the regulation of synaptic function.

## Results

### Nanoscale Imaging of the AZ Structure

**Figure 1:**
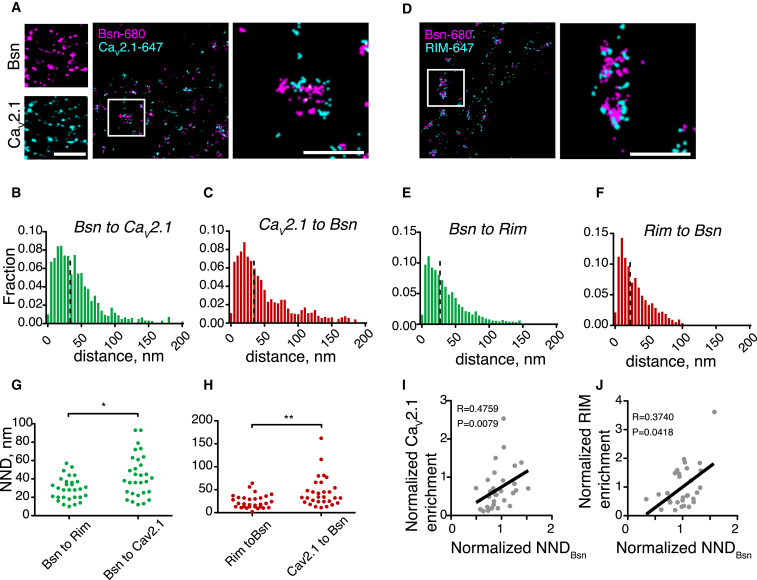
Dual-Color STORM Imaging of the AZ Structure (A) Imaging of Bsn and Ca_v_2.1. Left, Ca_v_2.1 and Bsn colocalize in puncta as visualized using confocal light microscopy. Scale bar, 5 μm. Right, neurons were stained for Bsn (CF680) and Ca_v_2.1 (AF647) and imaged using dual-color STORM. Zoomed regions, AZs in the “face-up” orientation. Scale bar, 1 μm. (B) Histogram of Bsn-to- Ca_v_2.1 NNDs in AZs. Dashed line denotes median value. (C) Histogram of Ca_v_2.1-to-Bsn NNDs in AZs. Dashed line denotes median value. (D–F) As in (A)–(C) but for RIM and Bsn. (G) Comparison of the median values between Bsn-to-Ca_v_2.1 and Bsn-to-RIM NNDs. (H) Comparison of the median values between Ca_v_2.1-to-Bsn and RIM-to-Bsn NNDs. ^∗^p < 0.05, ^∗∗^p < 0.01, Mann-Whitney test. (I) Correlation plot for relative AZ enrichment of Ca_v_2.1 versus Bsn-to-Bsn NND. (J) Correlation plot for relative AZ enrichment of RIM and Bsn-to-Bsn NND. n = 3, ten synapses/experiment.

Confocal microscopy imaging of Bsn and the canonical P/Q-type VGCC pore-forming subunit Ca_v_2.1 shows clear colocalization in punctate structures (Figure 1A), indicating that they both localize to the AZ; this level of resolution is, however, insufficient to visualize their detailed distribution within the AZ. To this end, we imaged Bsn and Ca_v_2.1 using dual-color direct stochastic optical reconstruction microscopy (dSTORM) (Winterflood et al., 2015) (Figures S1A–S1C).

Although Ca_v_2.1 and Bsn within the AZ were localized in close proximity, there was little spatial overlap between them, suggesting spatial segregation on the nanoscale (Figure 1A). The median nearest neighbor distance (NND) between Ca_v_2.1 and Bsn localizations within the AZ was 36.2 nm (Bsn to Ca_v_2.1) and 36.3 nm (Ca_v_2.1 to Bsn), with multiple instances of >100 nm distance (Figures 1B and 1C). Similar results were obtained for the distribution of Bsn and a release site marker RIM (Figures 1D–1F), although the median NNDs between Bsn and RIM localizations were consistently less than that between Bsn and Ca_v_2.1 (Figures 1G and 1H). To assess the relationship between Bsn clustering and Ca_v_2.1 recruitment, we plotted the AZ-specific enrichment of Ca_v_2.1 as a function of Bsn-Bsn NND. There was a positive correlation between these two values, indicating that loosening of Bsn clustering may be associated with Ca_v_2.1 accumulation (Figure 1I). Importantly, similar results were obtained for Bsn and RIM (Figure 1J). Together these data imply that Bsn clustering may limit the enrichment of other AZ proteins important for synapse function.

To assess the clustering and size of AZ protein domains, we binned the localization data into bins of 25, 50, and 100 nm and computed the Pearson’s correlation coefficients to measure the resulting spatial overlap (Figures S2A and S2B). As a positive control for overlap, we used samples that were doubly labeled for Bsn. At both 25- and 50-nm bins, overlap between Bsn and either Ca_v_2.1 or RIM was significantly different from Bsn-Bsn, while, at the 100 nm, all three labels showed nearly complete overlap (Figure S2C). Thus, Bsn and Ca_v_2.1 as well as Bsn and RIM are spatially segregated on a 25- to 50-nm scale, in agreement with the NND measurements and confocal microscopy data.

### Activity Blockade Unclusters the AZ Matrix

In contrast to a previous study reporting inactivity-induced decreases in Bsn levels in cortical neurons (Lazarevic et al., 2011), we found no evidence of such a decrease in hippocampal neurons (Glebov et al., 2016); furthermore, Bsn synthesis rate is independent of activity manipulation (Schanzenbächer et al., 2016), suggesting that activity-dependent regulation of the hippocampal AZ structure may differ from that in the cortex. We therefore used STORM to visualize the distribution of Bsn, Ca_v_2.1, and RIM following activity blockade.

**Figure 2:**
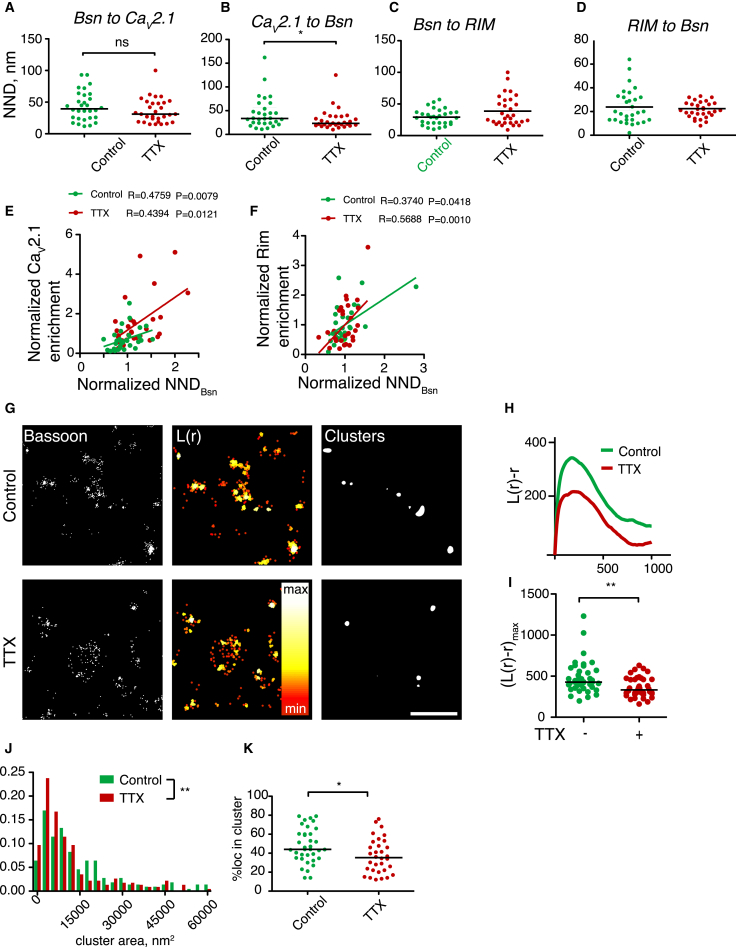
STORM Imaging Reveals the Effect of Activity Blockade on the AZ Structure (A) Bsn-to- Ca_v_2.1 NND is unaffected by TTX treatment. (B) Ca_v_2.1-to-Bsn NND is reduced by TTX treatment. (C) Bsn-to-RIM NND is unaffected by TTX treatment. (D) RIM-to-Bsn NND is unaffected by TTX treatment. (E) The correlation between Bsn-Bsn NND and Ca_v_2.1 enrichment with or without TTX treatment. (F) The correlation between Bsn-Bsn NND and RIM enrichment with or without TTX treatment. (G) Single-color STORM imaging of Bsn clustering and quantitative clustering analysis. Left, representative STORM images. Middle, cluster maps generated from local point-pattern analysis. Right, thresholded cluster maps. Scale bar, 1 μm. (H) Ripley’s L-function plots for the regions of interest depicted in (G). (I) The maximum of the Ripley’s L-function is reduced by TTX treatment. ^∗∗^p < 0.01, Mann-Whitney test. (J) The median area of the Bsn cluster is reduced by TTX. ^∗∗^p < 0.01, Mann-Whitney test. (K) The proportion of Bsn within the clusters is reduced by ^∗^p < 0.05, Student’s t test. n = 3, ten synapses/condition/experiment.

Blockade of action potential firing with tetrodotoxin (2 μM, 48 hr) had no effect on the number of Bsn and RIM localizations in the AZ; on the other hand, the number of Ca_v_2.1 localizations was increased (Figure S1D; data not shown), in agreement with previous studies (Lazarevic et al., 2011, Zhao et al., 2011) (also see Figure 4). Surprisingly, the corresponding NND measures were largely unchanged (Figures 2A–2D), with the exception of the slightly decreased Ca_v_2.1-Bsn NND, consistent with an increase in Ca_v_2.1 recruitment. Furthermore, the relationship between Bsn NND and either Ca_v_2.1or RIM recruitment, as well as the correlation between binned datasets, were not affected by tetrodotoxin (TTX) treatment (Figures 2E, 2F, and S2D). Thus, we conclude that despite the apparent AZ enrichment of VGCCs after activity blockade, the degree of spatial segregation between Bsn- and VGCC- or RIM-enriched domains is independent of neuronal activity.

For a quantitative insight into the activity-dependent changes in the AZ structure, we used clustering analysis based on Ripley’s K-statistics (Williamson et al., 2011) (Figures 2G–2K). Extraction of clustering statistics from thresholded data revealed that approximately 42% of Bsn labeling was organized in significantly smaller clusters than an average AZ (Schikorski and Stevens, 1997) (Figure 2J), indicative of the non-random distribution of Bsn within the AZ (Tang et al., 2016). TTX treatment decreased the maximal value of the Ripley function (Figures 2H and 2I), the area of clusters and the proportion of Bsn molecules in clusters (Figures 2J and 2K). In contrast, the density of Bsn within the cluster, the overall levels of Bsn and cluster number remained unchanged (Figures S1D–S1F). This suggests that blockade of neuronal activity leads to dissociation of Bsn from the clusters within the AZ, resulting in their shrinkage.

### Ratiometric Imaging of Protein Clustering

**Figure 3:**
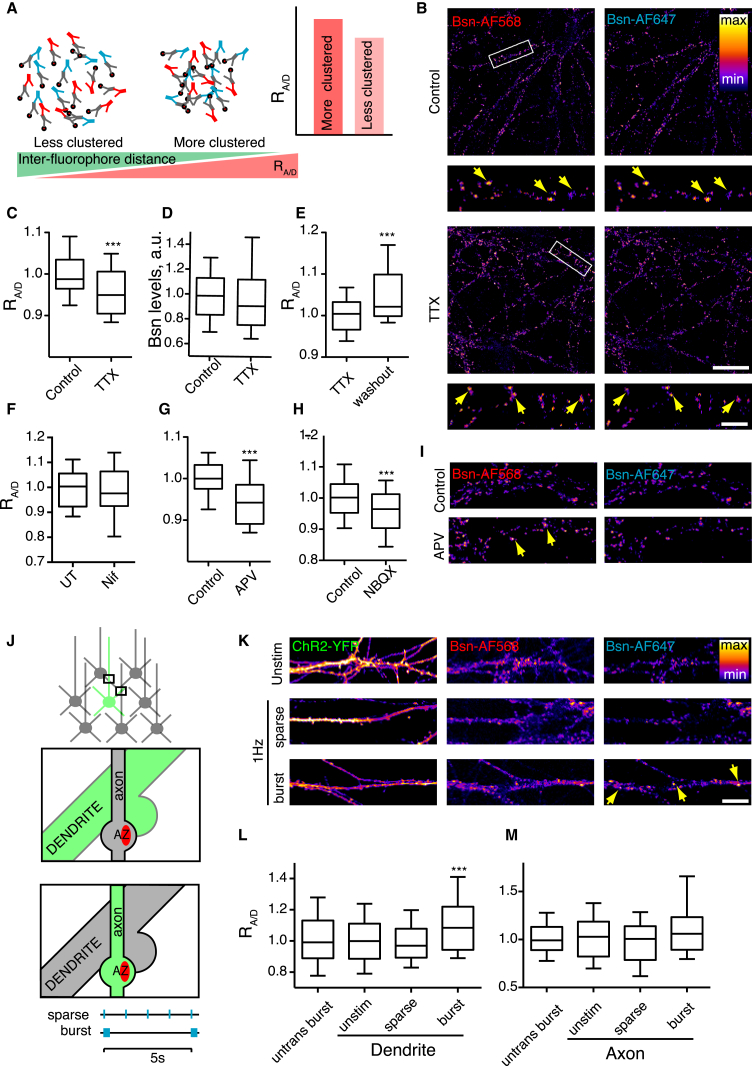
Ratiometric Imaging Reveals Activity-Dependent AZ Clustering on the Nanoscale (A) Schematic of the experimental approach; see Results and Experimental Procedures for detailed explanation. (B) Ratiometric imaging of TTX-treated neurons stained for Bsn. Arrows indicate AZs with the R_A/D_ representative for the condition. Scale bars, 20 μm (whole images) and 5 μm (magnified regions). (C) Cumulative data for (B); n = 6 experiments, 15 regions of interest (ROIs)/experiment. ^∗∗∗^p < 0.001, Student’s t test. (D) Bsn levels were unaffected by TTX treatment. (E) TTX washout reverses the R_A/D_ decrease. TTX, 96 hr TTX; washout, 48 hr TTX + 48 hr washout. n = 3, 15 regions of interest/experiment. ^∗∗^p = < 0.001, Student’s t test. (F) L-type VGCC blocker Nifedipine (2 μM, 48 hr) has no effect on R_A/D._ n = 3. (G and H) 48-hr treatment with 50 μM NMDAR blocker APV (G) and 10 μM AMPAR blocker NBQX (H) reduce R_A/D_. (I) Ratiometric imaging of APV-treated neurons stained for Bsn. Arrows indicate AZs with R_A/D_ representative for the condition. (J) Schematic of the optogenetic experimental setup. Top, sparse transfection of cultured neurons with ChR2-YFP. Second from top, rationale for identification of the *trans*-activated synapses formed between YFP-positive dendrites (green) and YFP-negative presynaptic boutons (gray). Third from top, rationale for identification of the *cis*-activated synapses formed between YFP-negative dendrites (gray) and YFP-positive presynaptic boutons (green). Bottom, two patterns used for activation of ChR2. Sparse, 1-Hz flashes. Burst, five flashes at 20 Hz every 5 s. (K) Cultures expressing ChR2-YFP were stimulated for 48 hr and fixed and stained for R_A/D_. Unstim, unstimulated culture; sparse and burst, see above. Shown are representative images of YFP-expressing dendrites with adjacent Bsn-positive AZs. Scale bar, 20 μm. (L) Quantification of the effect of ChR2 stimulation in *trans* on R_A/D_ in individual AZs. One AZ was defined as a single Bsn-positive punctum. (M) Same as in (L) but for stimulation in *cis*. ^∗∗∗^p < 0.001 compared to untransfected burst, one-way ANOVA with Kruskal-Wallis post test. n = 4 experiments, 10–30 regions of interest/experiment. Error bars indicate 10–90 percentile range.

The long image acquisition times and substantial computational trade-offs associated with STORM imaging limit its use for investigation of large synapse populations. To circumvent these restrictions, we sought for an alternative approach allowing for rapid and high-throughput comparative visualization of protein clustering across multiple regions of interest, using ratiometric fluorescence resonance energy transfer (FRET) (Glebov and Nichols, 2004). In this approach (see Experimental Procedures for details), changes in distances between proteins can be visualized as changes in the ratio of intensities of the donor-acceptor fluorophores (Glebov and Nichols, 2004); we will hereafter refer to this as the Acceptor/Donor Ratio (R_A/D_) (Figure 3A).

To test the validity of this approach, we carried out the following control experiments (Figure S3). First, we performed ratiometric labeling using serial dilution of a primary anti-Bsn antibody, reasoning that increasingly diluted antibody concentrations should result in lower R_A/D_ values due to the increased inter-fluorophore distances. Indeed, dilution of the secondary antibodies progressively lowered the R_A/D_ (Figure S3A). Similar results were obtained for two other proteins, namely an endosome marker transferrin receptor (Figure S3B) and a synaptic vesicles protein vGlut1 (data not shown), highlighting the generalizable nature of the ratiometric approach. Second, to demonstrate that our approach can report acute decreases in clustering of a probe, we ratiometrically imaged limited actin depolymerization in neurons using a low concentration (500 nM) of the actin depolymerizing drug Latrunculin A (LatA) that does not reduce the overall levels of the polymerized filamentous (F)-actin (Glebov et al., 2015). Under these conditions, a decrease in R_A/D_ was readily detected, consistent with decreased clustering of F-actin (Figure S3C). Third, to show that our assay can detect acute increases in clustering, we used a recombinant cell-surface glycosylphosphatidylinositol (GPI)-GFP probe after inducing clustering by antibody crosslinking (Glebov and Nichols, 2004). 10 min crosslinking resulted in a visible clustering of the probe, concomitant with a robust increase in R_A/D_ (Figure S3D). Taken together, these controls show that the ratiometric approach can be used to report nanoscale changes in clustering of a probe.

### Ratiometric Imaging Confirms Inactivity-Induced AZ Bsn Matrix Unclustering

Having validated our assay, we used it to study the activity-dependent nanoscale changes of the AZ matrix using confocal microscopy. Bsn levels and the size of the Bsn-positive puncta were unaffected by TTX, suggesting that the gross morphology of the AZ remained intact (Figures 3D and S3E). However, in agreement with the STORM data (Figure 2), TTX treatment led to a decrease in R_A/D_ for Bsn (Figures 3B and 3C). An R_A/D_ decrease was also observed using two alternative anti-Bsn antibodies, further confirming the unclustering of the AZ matrix (Figure S3F). The FRET ratio measured using the sensitized emission approach (Glebov and Nichols, 2004) was also reduced by TTX treatment (Figure S4A). In contrast to Bsn, R_A/D_ values for the postsynaptic matrix proteins Psd95 and Gephyrin remained unchanged by the TTX treatment (Figures S4C and S4D), in agreement with recent studies showing only subtle local effects of activity modulation on postsynaptic scaffolding (MacGillavry et al., 2013, Specht et al., 2013, Tang et al., 2016).

A washout (48 hr) of TTX led to an increase in R_A/D_ (Figure 3E), indicating that the effect was reversible. The effect of TTX was evident after 24 hr, but not 3 hr (Figure S4E; data not shown), consistent with a typical timeline for homeostatic synaptic plasticity (Pozo and Goda, 2010). R_A/D_ was not significantly altered by TTX when a spectrally non-overlapping pair of fluorophores was used (Figure S3G), indicating that the observed change in R_A/D_ could be solely attributed to a change in FRET efficiency, rather than a change in the binding efficiency of the antibody.

To directly compare the ratiometric assay with the STORM measurement, we employed our data from the serial dilution experiment (Figure S3A) to plot the relationship between R_A/D_ and the inter-fluorophore distance. Ultrastructural investigations of AZ have shown that the AZ architecture is essentially flat, with the AZ plane immediately underlying the presynaptic membrane by the synaptic cleft (Harris and Weinberg, 2012, Meyer et al., 2014, Murthy et al., 2001, Südhof, 2012, Tang et al., 2016). According to the constraints of the two-dimensional approximation of the AZ structure, the distance between the fluorophores (i.e., labeled Bsn molecules) within the AZ matrix should scale proportionally to the square root of the dilution factor (Figure S3H). In agreement with this, the decrease in R_A/D_ plotted against the estimated increase in distance was well fitted by a single exponential decay curve. The decrease in Bsn R_A/D_ measured following the TTX treatment (4.5%) matched the increase in Bsn-Bsn NND measured using STORM (33.7%) (Figure S3H) directly confirming the validity of the ratiometric approach for visualization of nanoscale clustering.

Having established that blockade of activity results in a decrease in AZ clustering, we tested the converse, i.e., whether enhanced activity would result in an increase in AZ clustering. To this end, we took advantage of an optogenetic stimulation paradigm that allows for controlled induction of neuronal activity in both space and time. Neurons expressing Channelrhodopsin2-YFP (ChR2-YFP) were stimulated with two previously characterized “burst” and “sparse” stimulus patterns (Grubb and Burrone, 2010). To quantify the effect of activation in *cis*, we selected the synapses formed by the ChR2-expressing axons onto the untransfected dendrites. Conversely, to quantify the effect of activation in *trans*, R_A/D_ was measured in the synapses formed by untransfected axons onto the ChR2-expressing dendrites (Figure 3J). Only “burst” stimulation in *trans* resulted in an increase in R_A/D_ (Figures 3K and 3L), while R_A/D_ in distal synapses was unaffected by the stimulation (Figure 3M). Specific patterns of neuronal activity therefore drive transsynaptic AZ clustering in a localized manner.

### NMDAR Activity and Cannabinoid Signaling Regulate AZ Matrix Clustering

A major consequence of postsynaptic depolarization is an increase in postsynaptic Ca^2+^ influx, which in hippocampal neurons is primarily mediated by L-type VGCCs and NMDA-type glutamate receptors (NMDARs) (Bloodgood and Sabatini, 2007). To establish their contributions to the regulation of AZ clustering, we incubated neurons in either the L-type VGCCs blocker nifedipine or the NMDAR antagonist (2R)-amino-5-phosphonovaleric acid (APV). APV, but not nifedipine, resulted in a significant decrease in R_A/D_, showing that NMDAR activation was required for AZ matrix clustering (Figures 3F and 3G). This effect was recapitulated by blockade of the AMPA/kainate-type glutamate receptors (AMPAR/KAR) with 2,3-dihydroxy-6-nitro-7-sulphamoyl-benzo(f)quinoxaline-2,3-dione (NBQX), further confirming the role of postsynaptic depolarization in AZ clustering (Figure 3H). Neither APV nor NBQX treatment affected the levels of Bsn (Figure S4B). The timescale of these effects (Figure S4E; data not shown) was consistent with the timescale of slow synaptic scaling (Pozo and Goda, 2010) rather than that of a rapid brain-derived neurotrophic factor (BDNF)-dependent homeostatic regulation of presynaptic activity (Jakawich et al., 2010).

To further investigate the signaling mechanisms linking postsynaptic activity with AZ matrix clustering, we tested for the involvement of endocannabinoid signaling, which is a major activity-dependent retrograde pathway operating in hippocampal neurons (Castillo et al., 2012). Inhibition of endocannabinoid synthesis by tetrahydrolipstatin (THL) resulted in a decrease in R_A/D_, as did bidirectional manipulation of cannabinoid signaling by a cannabinoid receptor 1 (CB1) agonist arachidonyl-2′-chloroethylamide (ACEA) and an inverse agonist AM251 (Figures S4F–S4I). These data implicate NMDAR activation and endocannabinoid release as two mechanisms involved in transsynaptic regulation of AZ matrix clustering.

### Inactivity-Induced Presynaptic Recruitment of Multiple AZ Proteins

**Figure 4:**
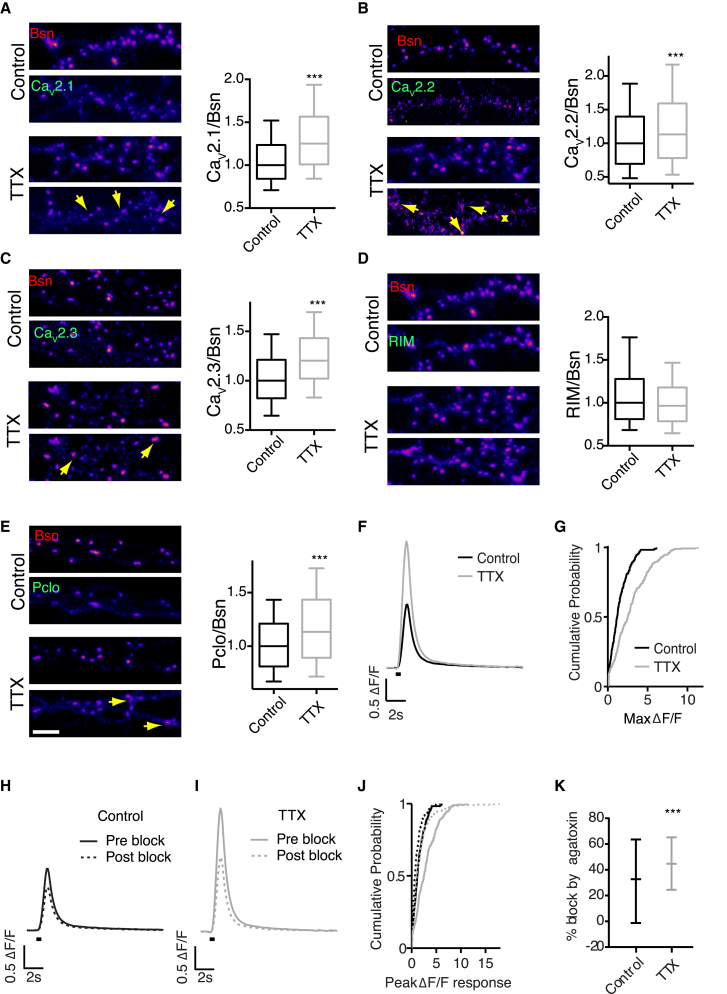
Activity Blockade Leads to Recruitment of Multiple AZ Proteins in Hippocampal Neurons and Upregulation of Presynaptic Ca^2+^ Influx through P/Q-type VGCCs (A) TTX treatment (2 μM, 48 hr) results in AZ enrichment of Ca_v_2.1. n = 3. (B) TTX treatment results in AZ enrichment of Ca_v_2.2. Arrows indicate individual AZs; asterisk denotes a Ca_v_2.2 punctum not associated with AZ. n = 3. (C) TTX treatment results in AZ enrichment of Ca_v_2.3. n = 3. (D) TTX treatment does not affect AZ enrichment of RIM. n = 3. (E) TTX treatment results in AZ enrichment of Pclo. n = 4. (F and G) Increase in presynaptic Ca^2+^ signaling following the TTX blockade. Neurons transfected with a Ca^2+^ sensor SyGCaMP6f were stimulated with ten APs at 20 Hz. For 48 hr prior to imaging neurons were incubated in either untreated media (Control) or media containing 2 μM TTX. ΔF/F responses were increased after TTX treatment. (F) Mean ΔF/F response traces (n = 224 synapses for control, n = 265 synapses for TTX, stimulus period indicated by black bar). (G) Cumulative distribution of peak ΔF/F responses. n = 4. (H–K) P/Q VGCCs are preferentially recruited to the AZ following activity blockade. (H) Mean ΔF/F response traces before (solid line) and after (dashed line) blockade of P/Q channels with 100 nM ω-agatoxin IVA in control neurons for the same synapses. (I) Same as (H) for TTX-treated neurons. (J) Cumulative distribution of synaptic peak ΔF/F responses in all conditions. (K) Percentage block by agatoxin is increased in TTX-treated cells, indicating a higher proportion of the Ca^2+^ influx mediated by P/Q-type VGCCs. Data shown as median + IQR. ^∗∗^p = 0.0032, Mann-Whitney U test. Error bars indicate 10–90 percentile range.

Given the discrepancy between our findings and the effects of activity blockade on AZ composition in cortical neurons (Lazarevic et al., 2011), we investigated the effect of activity blockade on presynaptic recruitment of other AZ proteins in the hippocampal synapse. In agreement with our STORM data, there was a significant increase in Ca_v_2.1 channel (P/Q-type VGCCs) recruitment (Figure 4A). In contrast, synaptic levels of RIM were unchanged (Figure 4D), as were those of another adaptor AZ protein Munc13-2 (data not shown). In yet another contrast with cortical neurons, synaptic recruitment of a Bsn-related AZ scaffolding protein Piccolo (Pclo) was also increased (Figure 4E), as were the levels of two other presynaptic VGCCs, R-, and N-type (Figures 4B and 4C). Taken together, these data are consistent with the notion that the decreased clustering of the AZ matrix may allow for increased recruitment of presynaptic machinery in hippocampal neurons.

To obtain a functional measure of the TTX effect on presynaptic Ca^2+^ signaling, we measured AP-evoked calcium influx in presynaptic terminals with the genetically encoded presynaptic calcium indicator SyGCaMP6f. In agreement with the structural findings, we observed a strong increase in calcium influx following 48-hr incubation in TTX (Figures 4F and 4G). We then used a specific channel blocker ω-Agatoxin-IVA (Aga) to quantify the contribution of the P/Q-type VGCCs to presynaptic Ca^2+^ rise in untreated and TTX-treated cultures. Inhibition of the SyGCaMP6f signal by Aga was significantly increased by TTX blockade (44% versus 32%, p = 0.0032) (Figures 4H–4K). Thus, activity blockade results in a preferential recruitment of P/Q-type VGCCs to the synapse.

### Synapse-Specific Correlation between AZ Matrix Clustering and Presynaptic Function

**Figure 5:**
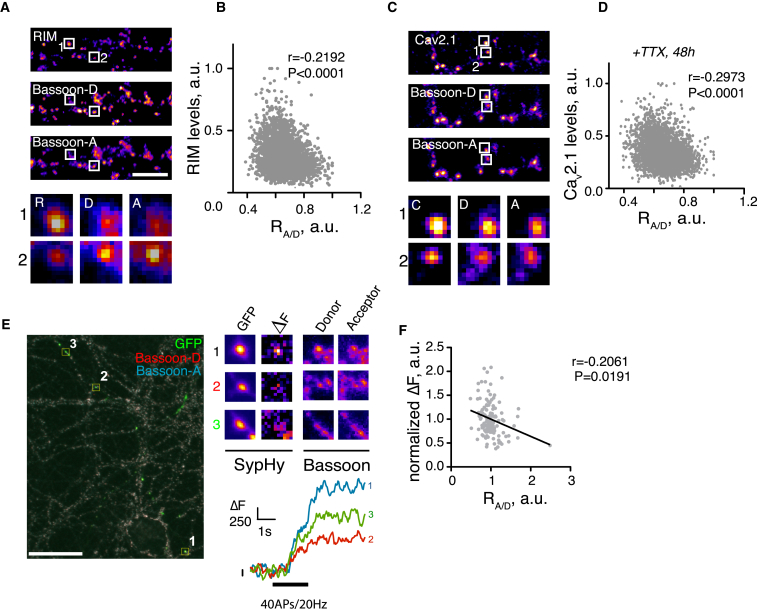
Recruitment of Presynaptic Machinery and Synaptic Vesicles Cycling Negatively Correlate with R_A/D_ (A) Neurons were fixed and stained for RIM with AF-405 and Bsn with AF-568 and AF-647. Arrows depict multiple puncta of RIM-Bsn colocalization, likely corresponding to individual AZs. Scale bar, 10 μm. (B) Synapse-specific correlation between RIM levels and R_A/D_. n = 4. (C) Neurons were treated with TTX for 48 hr, fixed, and stained for Ca_v_2.1 with AF-405, Bsn with AF-568 and AF-647. Scale bar, 10 μm. (D) Quantification of (A). n = 3. (E) Example of an overlaid image combining live imaging of SypHy and fixed ratiometric imaging of Bsn. Left, an entire field of view with three regions of interest selected. Scale bar, 20 μm. Top right, magnified images of the three chosen regions of interest, showing respective intensities for GFP and Bsn. Bottom right, live imaging traces for the three chosen regions of interest. (F) Correlation between presynaptic cycling as measured by SypHy imaging and R_A/D._ n = 5. r, Spearman’s rank correlation coefficient.

To explore the functional relevance of AZ clustering regulation, we hypothesized that AZ clustering may correlate with recruitment of other presynaptic proteins involved in neurotransmitter release. To test for this, we measured enrichment of RIM and Ca_v_2.1 in individual AZs and plotted it against local R_A/D_. RIM enrichment was negatively correlated with R_A/D_ (Figures 5A and 5B), showing that the AZs with looser clustering preferentially recruited RIM, in agreement with our STORM data (Figures 1 and 2). In contrast, there was no correlation between R_A/D_ and the levels of a ubiquitous pre/postsynaptic scaffolding protein CASK (Figure S5B) (Hsueh et al., 1998, Südhof, 2012). There was also no correlation between R_A/D_ and levels of Ca_v_2.1, possibly reflecting the existence of an extrasynaptic Ca_v_2.1 pool likely to obfuscate a presynaptic specific correlation when performed at this level of resolution (Figure S5A); indeed, in our cells accumulation of the Ca_v_2.1 label in the dendritic shaft and the cell body was often observed, and ultrastructural evidence supports the presence of the extrasynaptic Ca_v_2.1 pool (Indriati et al., 2013). Presynaptic enrichment of Ca_v_2.1 at the AZ by TTX treatment, however, led to emergence of an inverse correlation with Bsn R_A/D_ (Figures 5C and 5D), indicating that Ca_v_2.1 was preferentially recruited to the AZs with less Bsn clustering, in agreement with the STORM data (Figures 1 and 2). The levels of all of the above strongly correlated with local Bsn levels, indicating that the bigger synapses contained more of synaptic proteins (Figures S5C–S5F).

To investigate the link between AZ matrix clustering and presynaptic function, we took advantage of the two well-established assays for synaptic vesicle cycling based on (1) uptake of an antibody against the extracellular/luminal domain of the synaptic vesicles protein Synaptotagmin 1 (Scheiffele et al., 2000) and (2) live imaging of a fluorescent GFP-based pH-sensitive synaptic vesicles probe SypHy (Miesenböck et al., 1998). AZ-specific R_A/D_ exhibited a negative correlation with the uptake of anti-Syt1 antibody under conditions favoring either spontaneous (20 min at 37°C in presence of 2 μM TTX) or evoked (4 min at 37°C in presence of 50 mM KCl) presynaptic release (Figures S6A and S6B).

Additionally, we combined live SypHy imaging of presynaptic vesicle cycling with post hoc ratiometric imaging of AZ clustering to directly correlate the functional readout from the individual synaptic boutons with the structure of the individual AZs (Figure S6C). Imaging of presynaptic function with SypHy also yielded a negative correlation between R_A/D_ and the size of the rapidly releasable pool, assessed functionally from the amplitude of the response to a stimulus of 40 APs delivered at 20 Hz frequency (Figures 5E and 5F). The extent of local AZ matrix clustering is therefore inversely correlated with recruitment of presynaptic release machinery and synaptic vesicle cycling.

### Presynaptic Actin Dynamics Regulate AZ Clustering

To further characterize the mechanism underlying activity-dependent AZ clustering dynamics, we focused on actin dynamics that have been previously implicated in regulation of presynaptic plasticity (Cingolani and Goda, 2008, Morales et al., 2000, Sankaranarayanan et al., 2003). Actin dynamics have been previously suggested to act as a restrictive influence on presynaptic release through curbing of synaptic vesicles cycling (Morales et al., 2000). Thus, actin dynamics represent a promising candidate mechanism for linking neuronal activity and synaptic structure.

**Figure 6:**
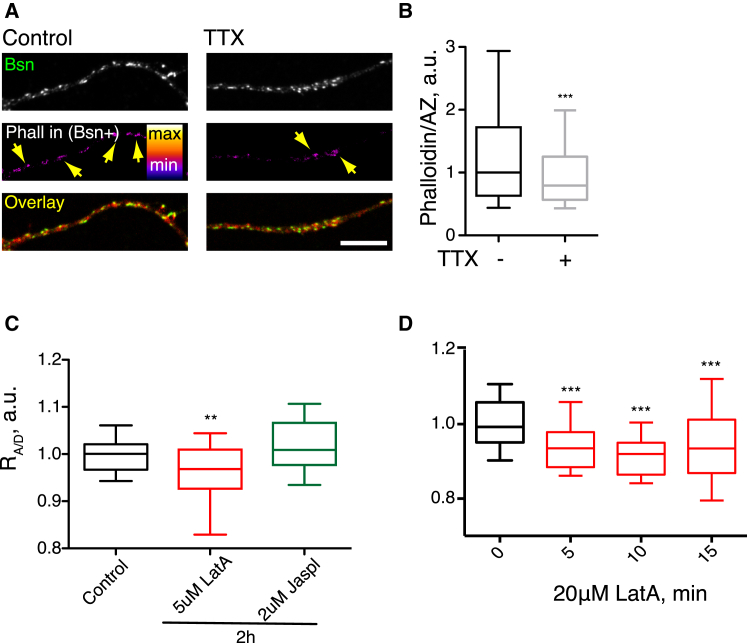
Activity-Dependent Actin Dynamics Regulate AZ Clustering (A) Synaptic F-actin levels are regulated by activity. Neurons were treated with TTX for 48 hr and stained for Bsn and AF647-Phalloidin. Second row depicts Phalloidin signal present in Bsn-positive puncta (AZs). (B) The intensity of Phalloidin staining in Bsn-positive puncta in control and TTX-treated cultures. One region of interest corresponds to one Bsn-positive punctum. n = 3, 310–806 regions of interest/experiment. (C) The effect of 2-hr incubation with 5 μM actin-depolymerizing drug Latrunculin A (LatA) or 2 μM actin-polymerizing drug Jasplakinolide (Jaspl) on R_A/D_. n = 4, 15 regions of interest/experiment. (D) Depolymerization of actin by 20 μM LatA rapidly decreases R_A/D_. Error bars indicate 10–90 percentile range.

We first assessed the effect of long-term activity blockade on actin levels. TTX treatment globally reduced the F-actin levels in the entire neuron, suggesting that actin polymerization was regulated by the network activity (data not shown). Specifically, the levels of F-actin present in Bsn-positive puncta were reduced, consistent with inactivity-induced depolymerization of synaptic F-actin (Figures 6A and 6B). In agreement with this, prolonged pharmacological actin depolymerization by 5 μM LatA for 2 hr decreased Bsn R_A/D_; in contrast, block of actin depolymerization with Jasplakinolide (Jaspl) had no effect on Bsn R_A/D_ (Figure 6C). On the shorter timescale, treatment with 20 μM LatA decreased Bsn R_A/D_ within 5 min, consistent with the previously reported rapid induction of presynaptic plasticity by this actin depolymerization (Morales et al., 2000); at the same time, the area of the AZ remained the same, suggesting that the structural rearrangement was restricted to the nanoscale (Figure 6D). We propose that actin dynamics are a possible candidate for regulating AZ composition through activity-dependent remodeling of the AZ scaffolding.

## Discussion

In this study, we have combined super-resolution, ratiometric, and functional imaging to establish the link between activity, AZ matrix organization, recruitment of presynaptic release machinery and synapse function at an archetypal CNS synapse. Crucially, we show that the AZ structure belies a surprising potential for reversible bidirectional reorganization at the nanoscale level, controlled by local postsynaptic activity that, in turn, fine-tunes presynaptic function. Taken together, our results show that local neuronal activity dynamically controls AZ organization through actin dynamics to modulate presynaptic structure and function.

### AZ Architecture on the Nanoscale and Its Regulation by Activity

Our STORM data show that the non-random organization of the AZ matrix characterized by Bsn clustering shows little overlap with the VGCCs and RIM-positive domains, with spatial segregation on the scale of 25–50 nm. These observations echo the previously reported differences in the distribution of the structural AZ components and VGCCs at the *Drosophila* neuromuscular junction (Ehmann et al., 2014, Fouquet et al., 2009), suggesting that the existence of distinct structural domains may be a core feature of the AZ structure.

Our data also reveal an activity-dependent remodeling of the AZ at the nanoscale. Chronic blockade of network activity results in a decrease in the area of the Bsn clusters and a corresponding increase in Bsn NND, without any major changes in the overall AZ morphology or Bsn levels. These results agree with the previously published data in hippocampal neurons (Glebov et al., 2016, Schanzenbächer et al., 2016) but are at odds with another study in cortical neurons (Lazarevic et al., 2011). The reason for this discrepancy is not clear but may be due to the intrinsic differences between cell types. The activity-induced unclustering of the AZ matrix did not affect the distance between the Bsn domains and the neighboring VGCCs/RIM-positive areas, indicating sustained spatial segregation at the AZ following activity-dependent plasticity. This feature is in line with the idea that Bsn domains act to limit the spatial extent of their neighbors.

### Signaling Pathways Shaping Presynaptic Structure and Function

Our optogenetics data suggest the presence of a putative local mechanism linking postsynaptic activity with presynaptic structure and function. These data are further supported by the AZ unclustering observed following chronic NMDAR and AMPAR/KAR blockade and suggest the involvement of postsynaptic Ca^2+^ signaling. Further still, calcium influx through L-type VGCCs was not required, indicating that the mode of calcium influx is important and likely involves local synaptic routes. Indeed, the requirement for NMDAR activation fits with the canonical role of NMDARs as coincidence receptors of transsynaptic depolarization and neurotransmitter release (Hunt and Castillo, 2012). Taken together, these data establish another role for NMDARs in regulating presynaptic function.

The precise details of the downstream events activated by NMDAR activation will require further investigation. Although our pharmacological data are consistent with the role of cannabinoid signaling in regulating AZ clustering and neurotransmitter release, the actual regulatory mechanism is likely to be complex, given that both upregulation and blockade of CB1 resulted in an unclustering of the AZ matrix. Signaling through CB1 receptors is complex and includes feedbacks that downregulate receptor availability following sustained activity (Dudok et al., 2015), which could help explain the responses observed here. Furthermore, manipulation of cannabinoid signaling in the hippocampus has been shown to modulate NMDA receptor function (Hampson et al., 2011), suggesting cross-talk between excitatory neurotransmission and cannabinoid signaling.

### A Model for Integrating Synaptic Activity, Structure, and Function

**Figure 7:**
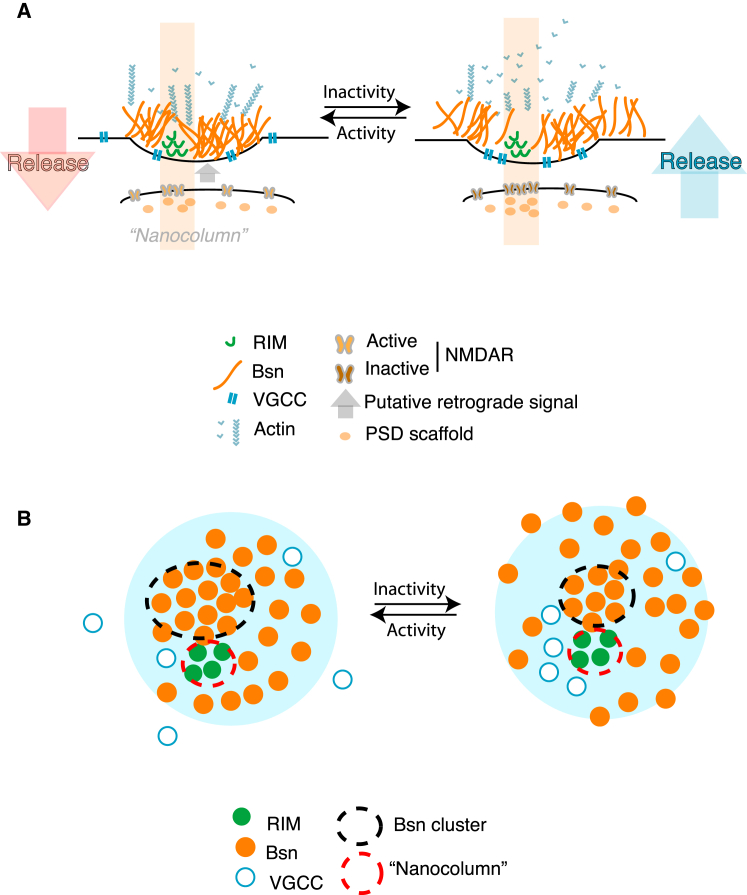
A Proposed Model for Local Integration of Postsynaptic Activity, Nanoscale Structure, and Function (A) Lateral view of the proposed synaptic organization. Left, postsynaptic NMDAR activity within the transsynaptic nanocolumn generates a putative retrograde signal that locally maintains the organization of the presynaptic clustering through ongoing actin polymerization. The clustering of the AZ matrix limits the recruitment of presynaptic machinery such as VGCC and RIM to the AZ, modulating presynaptic release. Right, decreased levels of postsynaptic activity reduce presynaptic AZ matrix clustering: this, in turn, reduces the extent of macromolecular congestion, facilitating ingress of presynaptic machinery molecules into the AZ site and thus enabling the inactivity-induced increase in Ca^2+^ influx and release probability. (B) En face view of the proposed AZ organization. Unclustering of the Bsn-rich matrix is associated with recruitment of VGCCs to the vicinity of the RIM-enriched release sites within the transsynaptic nanocolumns.

How does the dynamic organization of the AZ matrix control presynaptic function? On the basis of our data, a model can be proposed whereby the clustering of the AZ matrix limits the recruitment of the presynaptic machinery, through competition for space (Figure 7). This notion is supported by the following observations: (1) enduring activity-independent spatial segregation between AZ matrix and VGCCs/release sites (Figures 1 and 2); (2) opposite effects of activity blockade on AZ matrix clustering and recruitment of multiple AZ components (Figures 2, 3, 4, and 5); (3) AZ-specific negative correlation between matrix clustering and presynaptic machinery recruitment/synaptic function (Figures 1, 4, and 5); and (4) activity-dependent regulation of synaptic F-actin levels and rapid induction of AZ matrix unclustering by actin depolymerization (Figure 6).

The limiting effect of the AZ matrix scaffolding on presynaptic function is consistent with the evidence showing distinct subdomains within the *Drosophila* AZ (Ehmann et al., 2014, Fouquet et al., 2009), a proposed spatial segregation at the calyx of Held AZ (Nakamura et al., 2015), estimates of molecular crowding at the synapse (Wilhelm et al., 2014), and a role for actin polymerization as an activity-regulated presynaptic restrictive factor (Morales et al., 2000, Sankaranarayanan et al., 2003).

### Limitations of the Current Study

The modulatory (rather than mandatory) role for AZ scaffolding agrees well with the apparently non-essential role of Bsn and Pclo in synaptic transmission (Hallermann et al., 2010, Mukherjee et al., 2010), in contrast with the essential role of the other, less numerous components of the AZ (Betz et al., 2001, Kaeser et al., 2009, Schoch et al., 2002). Moreover, the restrictive modality of the AZ matrix appears to act as a functional counterpoint to the protein-protein interactions of other AZ proteins that serve to recruit presynaptic machinery to the AZ (Betz et al., 2001, Davydova et al., 2014, Kaeser et al., 2011, Südhof, 2012).

Certain considerations arising from the experimental techniques need to be taken into account when interpreting the results of this study. First, a combination of primary and secondary antibody labeling will affect the ability of STORM imaging to report on the true localization of the epitope in question. Second, the NND values must be considered in the context of the large size of the AZ proteins (Südhof, 2012) and lack of information about their mutual orientation; our understanding of the AZ organization therefore remains incomplete. Furthermore, it is important to point out that the nature of the ratiometric assay used here to measure protein clustering is indirect and based on labeling with primary and secondary antibodies that could introduce errors. However, the agreement between the ratiometric and the STORM data (Figure S3H) shows its applicability to samples of known topology. It is hoped that future studies of AZ organization will benefit from novel imaging tools, e.g., direct measurement of local macromolecular crowding (Boersma et al., 2015).

### Macromolecular Congestion as a Regulatory Mechanism at the Synapse and Beyond

Limiting presynaptic function by molecular congestion is in contrast to the permissive effect afforded by the clustering of the postsynaptic scaffold (MacGillavry et al., 2013, Specht et al., 2013, Tang et al., 2016), highlighting the fundamental differences in regulatory mechanisms operating on opposite sides of the synapse. From a purely mechanistic point of view, the curbing of presynaptic release by the AZ matrix could be viewed as an example of endogenous macromolecular congestion impacting on cellular function, with the clustering state of the AZ matrix restricting the ingress of presynaptic molecules into the AZ, thus limiting the composition of the functional AZ machinery. Similar principles have been proposed to modulate synaptic vesicles dynamics through congestion by the actin cytoskeleton (Morales et al., 2000) or collisions with organelles (Rothman et al., 2016). It is worth considering that, in a broader scope of cell biology, nanoscale structural plasticity of macromolecular assemblies may play a role in other functionally relevant contexts in the cell, e.g., in receptor signaling (James and Vale, 2012), endosome sorting (Wallrabe et al., 2007), and gene expression (Tan et al., 2013). Further understanding of this emerging regulatory modality will benefit from locally correlating nanoscale structural characterization of these systems with measurable functional outcomes.

## Experimental Procedures

Detailed experimental procedures and materials can be found in the Supplemental Information.

For ratiometric imaging, coverslips with neurons were fixed, permeabilized, and labeled for immunocytochemistry using antibodies conjugated to two different fluorophores. For optogenetic stimulation, primary neuronal cultures were sparsely transfected with ChR2-YFP and stimulated for 48 hr. For quantification of synapse-specific correlation, coverslips were processed three-color immunocytochemistry, and recruitment of presynaptic machinery to the individual Bsn-positive puncta was correlated with the local R_A/D_ values. For live imaging of presynaptic function, neurons were sparsely transfected with the vesicle cycling sensor CMV::SypHy or Ca^2+^ sensor SyGCaMP6F and subjected to field stimulation while imaging. Images were analyzed using MATLAB codes (MathWorks). For correlative live-fixed imaging of presynaptic structure and function, live images of SypHy responses were aligned with the fixed ratiometric images using a MATLAB routine (Figure S6). For STORM imaging, samples were fixed, permeabilized, stained for the proteins of interest, and imaged using either a commercially available N-STORM Nikon system or a custom-built setup as described before (Winterflood et al., 2015). Imaging was performed in objective-type near-total internal reflection fluorescence (TIRF) mode. An image-correlation-based drift correction was employed. All data analysis was performed in ImageJ and MATLAB. Statistical analysis was carried out using GraphPad Prism 6.0. Sample distribution was assessed using D’Agostino and Pearson’s omnibus normality test; to assess the significance of differences between datasets, Mann-Whitney test was used unless noted otherwise. Error bars indicate 10–90 percentile range. ^∗∗∗^p < 0.001, ^∗∗^p < 0.01, ^∗^p < 0.05.

## Author Contributions

O.O.G. designed and oversaw the project, performed the experiments, analyzed the data, and wrote the manuscript with input from other authors. R.E.J. carried out the electrophysiological stimulation experiments, analyzed the data, and contributed to the writing of the manuscript. C.M.W. carried out the dual-color STORM imaging, analyzed the data, and participated in the writing of the manuscript. D.M.O. performed the Ripley’s K-function clustering analysis and participated in the writing of the manuscript. E.A.B. analyzed the confocal microscopy data. P.D. oversaw the cannabinoid signaling experiments and participated in the writing of the manuscript. H.E. oversaw the dual-color STORM imaging experiments and participated in the writing of the manuscript. J.B. developed the registration algorithm, oversaw the project, and co-wrote the manuscript.

## Supplemental Information

Document S1. Supplemental Experimental Procedures and Figures S1–S6

Document S2. Article plus Supplemental Information
