# Supplementary material for: Nanoscale Structural Plasticity of the Active Zone Matrix Modulates Presynaptic Function
Source: Cell Rep. 2017 Mar 14;18(11):2715–28. doi: 10.1016/j.celrep.2017.02.064 (PMC5368346; doi:10.1016/j.celrep.2017.02.064)
Supplement: Document S1. Supplemental Experimental Procedures and Figures S1–S6 [file mmc1.pdf]

Cell Reports, Volume *18*

Supplemental Information

Nanoscale Structural Plasticity of the Active Zone Matrix Modulates Presynaptic Function

Oleg O. Glebov, Rachel E. Jackson, Christian M. Winterflood, Dylan M. Owen, Ellen A. Barker, Patrick Doherty, Helge Ewers, and Juan Burrone

Figure S1

Glebov&al

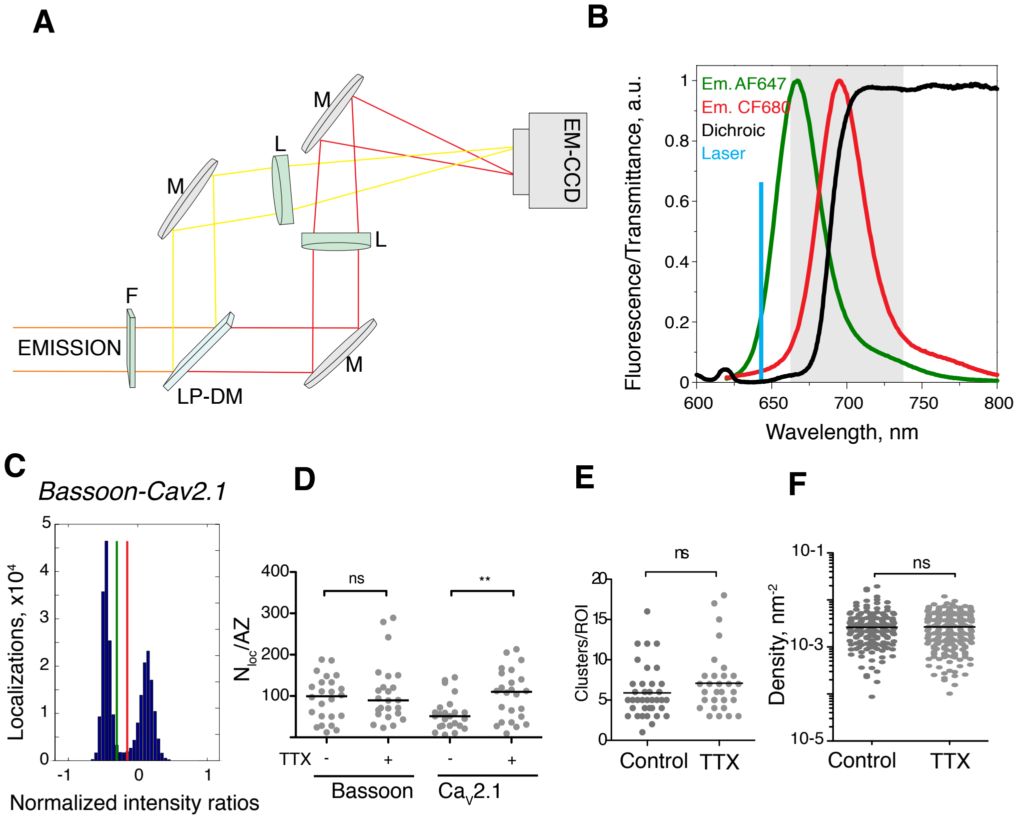

Figure S1. Supporting data for Figs. 1&2: STORM imaging of the AZ structure. (**A**), Schematic of the optical setup for dual color single-molecule localization microscopy by spectral-demixing dSTORM. F, emission filter; LP-DM, long-pass dichroic mirror (690 nm); M. mirrors; L, lenses; EM-CCD, Electron-multiplying charge-coupled device camera. (**B**) Emission spectra of AF647 (green) and CF680 (red), transmission of the dichroic (black) and transmission of the band-pass emission filter (gray box), and the 643 nm laser (blue). (**C**) Representative distributions of the normalized intensity ratios r=(Is–Il)/(Is+Il), where Is and Il are the fluorescence intensities of the short and long wavelength channels respectively. The green and red vertical lines delimit the assignment of the cut-off values. **(D-F**) Further effect of the TTX treatment on the AZ nanoscale structure; the number of Bsn and $Ca_V2.1$ localization events (LEs) per ROI (D), the number of Bsn clusters per 3x3μm ROI (E) and the density of Bsn LEs within the cluster (F). N=3.

Figure S2

Glebov&al

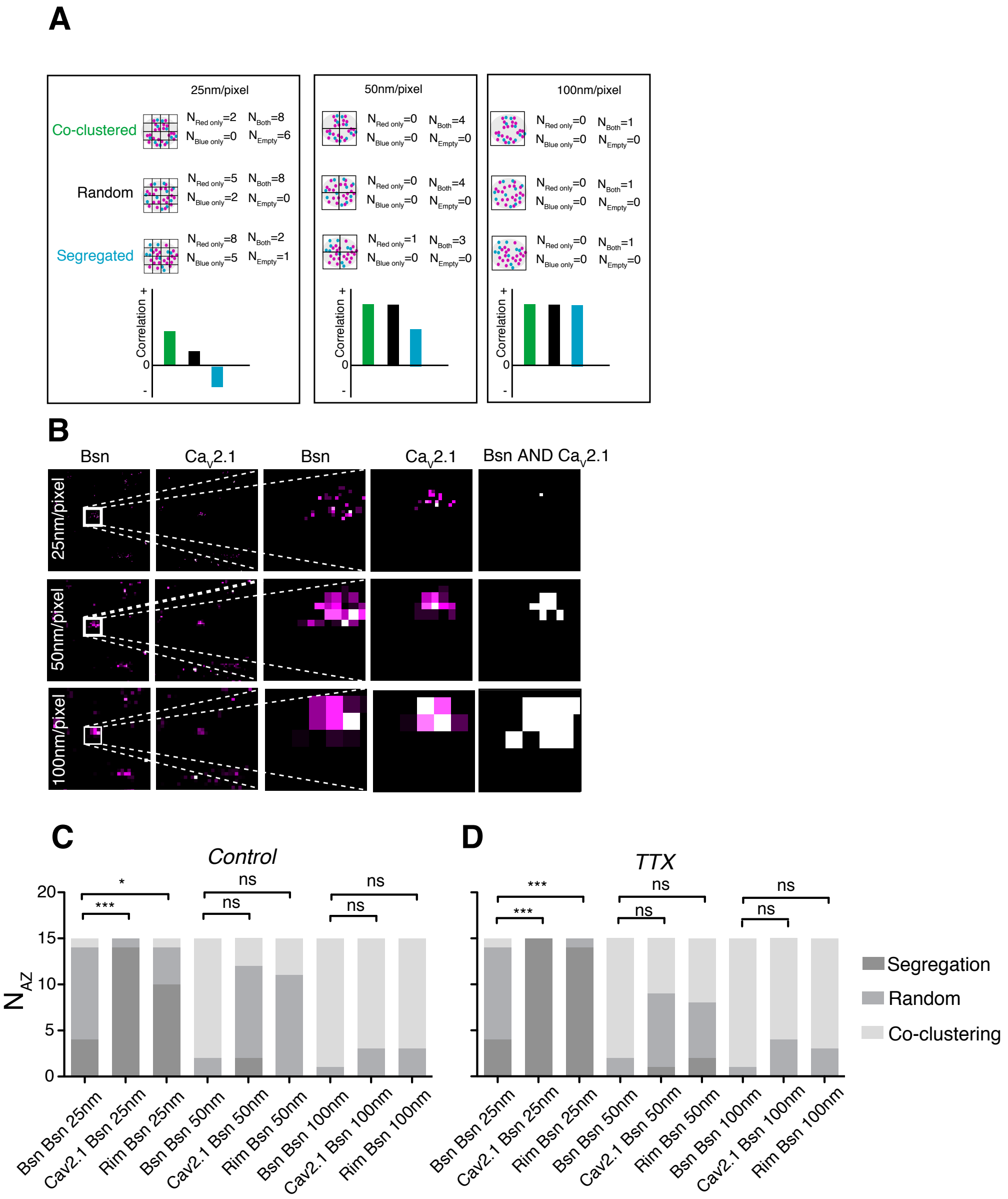

Figure S2. Supporting data for Fig. 1: Binning-based correlation analysis of spatial distribution of Bsn and Ca$_V$2.1. (**A**) Schematics of outcomes for differently distributed dually labeled AZ samples under 25nm/pixel, 50nm/pixel and 100nm/pixel binning conditions. As the binning capacity and the pixel size increases, correlations emerge in closely associated spatial arrangements. (**B**) An example of a binning procedure on a sample labeled for Bsn and Ca$_V$2.1. Note the emergence of pixels containing both Bsn and Ca$_V$2.1 (right, Bsn AND Ca$_V$2.1) in the larger bins; these will read out as positive correlation. (**C**) Bsn and Ca$_V$2.1 or RIM-containing pixels are spatially excluded under the 25nm binning regime as evidenced by the colocalization analysis. Segregation : significant negative correlation; Random: no significant correlation;Co-clustering, significant positive correlation. (**D**) Same as (C), but following TTX treatment. *p<0.05, *p<0.001, Fisher's exact test.

Figure S3

Glebov&al

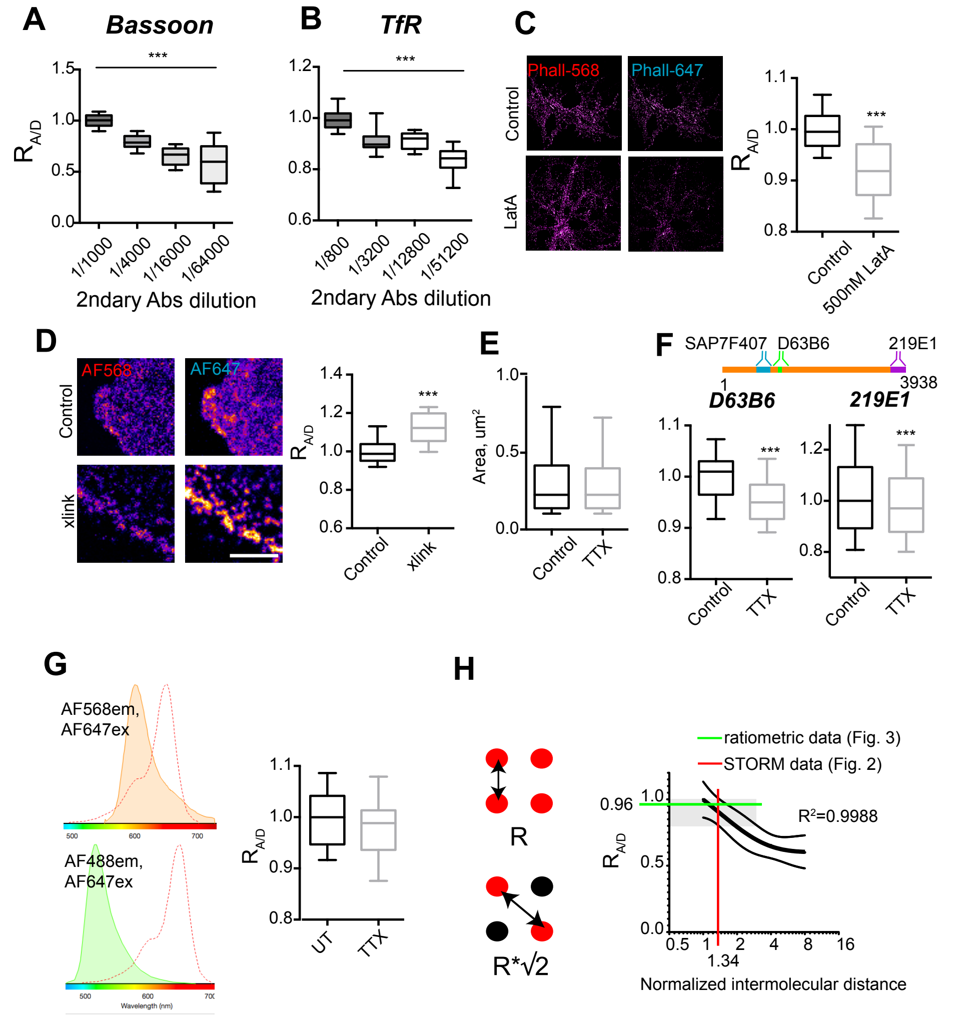

Figure S3. Supporting data for Fig. 3: Control experiments for the ratiometric imaging of protein clustering. **(A)** Control 1: serial dilution ratiometric staining of Bsn results in an increased distance between fluorophores and therefore decreased $R_{A/D}$. Neurons were stained with an anti-Bsn monoclonal mouse antibody and the mixture of donor- and acceptor-conjugated secondary anti-mouse antibodies. N=3. ***p<0.001, 1-way ANOVA. Error bars for all plots indicate 10-90 percentile range. **(B)** The same results were obtained for Transferrin receptor (TfR) N=3. ***p<0.001, 1-way ANOVA. **(C)** Control 2: partial depolymerization of F-actin results in an increased distance between actin filaments and therefore affects the $R_{A/D}$ measured through binding of an F-actin probe Phalloidin. Left, neurons were treated with 0.5μM Latrunculin A for 1h before fixation, permeabilization and staining with the mixture of donor- and acceptor-conjugated phalloidin. Note the apparently unchanged intensity of Phalloidin labeling in LatA-treated neurons(Glebov et al., 2015). Scale bar, 20μm. Right, quantification.***p<0.001, Student t-test. **(D)** Control 3: Acute induction of clustering of a cell surface protein results in a decrease in the donor-acceptor distance, manifesting itself in an increase in $R_{A/D}$. Left, U2OS cells expressing GPI-GFP were incubated with a rabbit anti-GFP antibody for 10min at RT, fixed, permeabilized, stained with a mouse anti-GFP antibody and a ratiometric mix of anti-mouse secondary antibodies. Scale bar, 5μm. Right, quantification. N=3. ***p<0.001, Student t-test. **(E)** TTX treatment did not affect the area of the Bsn-positive puncta. **(F)** Three different anti-Bsn antibodies demonstrate ratiometrically evident TTX-induced unclustering. Top, the schematic of the Bsn polypeptide chain charting the putative epitopes used by the three antibodies (to scale). Bottom left, unclustering of the Bsn matrix quantified by the ratiometric imaging involving the rabbit anti-Bsn monoclonal antibody D63B6. Bottom right, unclustering of the Bsn matrix quantified by the ratiometric imaging involving the mouse anti-Bsn monoclonal antibody 219E1. N=3. P<0.001, Mann-Whitney U test. **(G)** TTX treatment does not significantly change $R_{A/D}$ in the AF488-AF647 labeled cells (cf. Fig. 3C). Left, comparison of the overlap for the AF568 emission spectrum with AF647 excitation spectrum and AF488 emission spectrum with AF647 excitation spectrum. Note the almost complete lack of overlap in the latter case. **(H)** Direct comparison of the change in Bsn clustering measured using STORM and ratiometric analysis. Left, in a two-dimensional system, dilution of the labeling results in an increase in the distance proportional to the power of ½. Right, plot of the serial dilution data for Bsn, showing the single exponential fit and the 95% confidence intervals. The increase in distance as measured by STORM imaging (red line) corresponds to the decrease in $R_{A/D}$ measured ratiometrically (green line).

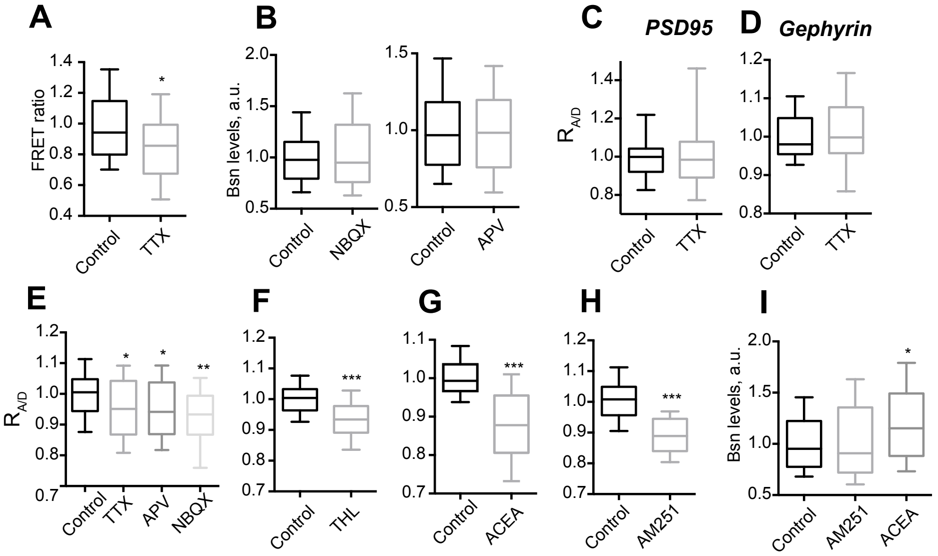

Figure S4. Supporting data for Fig. 3. (A) FRET ratio as measured by the sensitized emission method (Glebov and Nichols, 2004b) is decreased by TTX treatment. N=3, 15 ROIs/condition. **(B)** Bsn levels were not affected by APV and NBQX treatment. **(C-D)** $R_{A/D}$ values for postsynaptic matrix proteins Psd95 (C) and Gephyrin (D) were not affected by activity blockade. N=4, 15 ROIs/condition. **(E)** 24h treatment with TTX or APV or NBQX was sufficient to induce reduction of Bsn $R_{A/D}$. N=3 experiments, 15 ROI/condition. **(F)** 24h treatment with THL reduces $R_{A/D}$. N=3 experiments, 15 ROIs/condition. **(G)** Effect of 24h treatment with cannabinoid receptor agonist 10μM ACEA. N=4. **(H)** Effect of 24h treatment with cannabinoid receptor inverse agonist 4μM AM251. N=4. **(I)** Effect of ACEA and AM251 on Bsn levels. N=4, 15 ROI/condition. *p<0.05, ***p<0.001, Student t-test. Error bars indicate 10-90 percentile range.

Figure S5

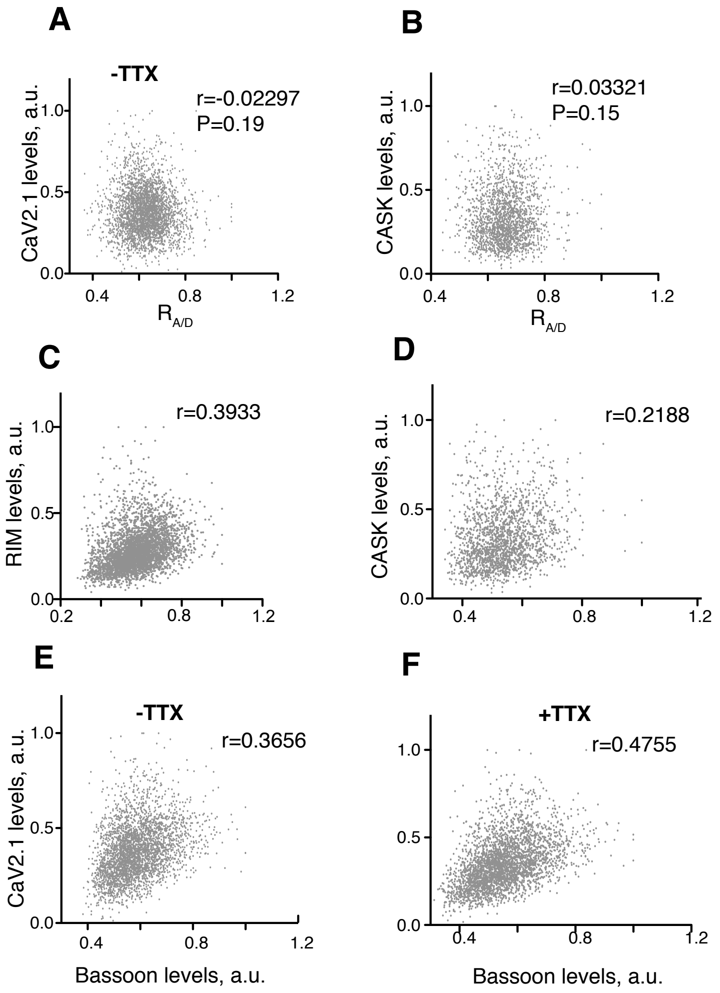

Figure S5. Supporting data for Fig. 5. Synapse-specific correlation between AZ clustering and presynaptic recruitment. (**A**) Lack of correlation between levels of $Ca_V2.1$and $R_{A/D}$ (compare with Fig. 5D). (**B**) Lack of correlation between levels of ubiquitous adapter protein CASK and $R_{A/D}$ in untreated cultures. (**C-F**) Levels of the following proteins were plotted against the levels of Bsn. (C) RIM. (D) CASK. (E) $Ca_V2.1$. (F) $Ca_V2.1$ (48h TTX). Datasets from Fig. 5 were used. r, Spearman's rank correlation coefficient. All correlations were significant (P<0.0001).

Figure S6

Glebov&al

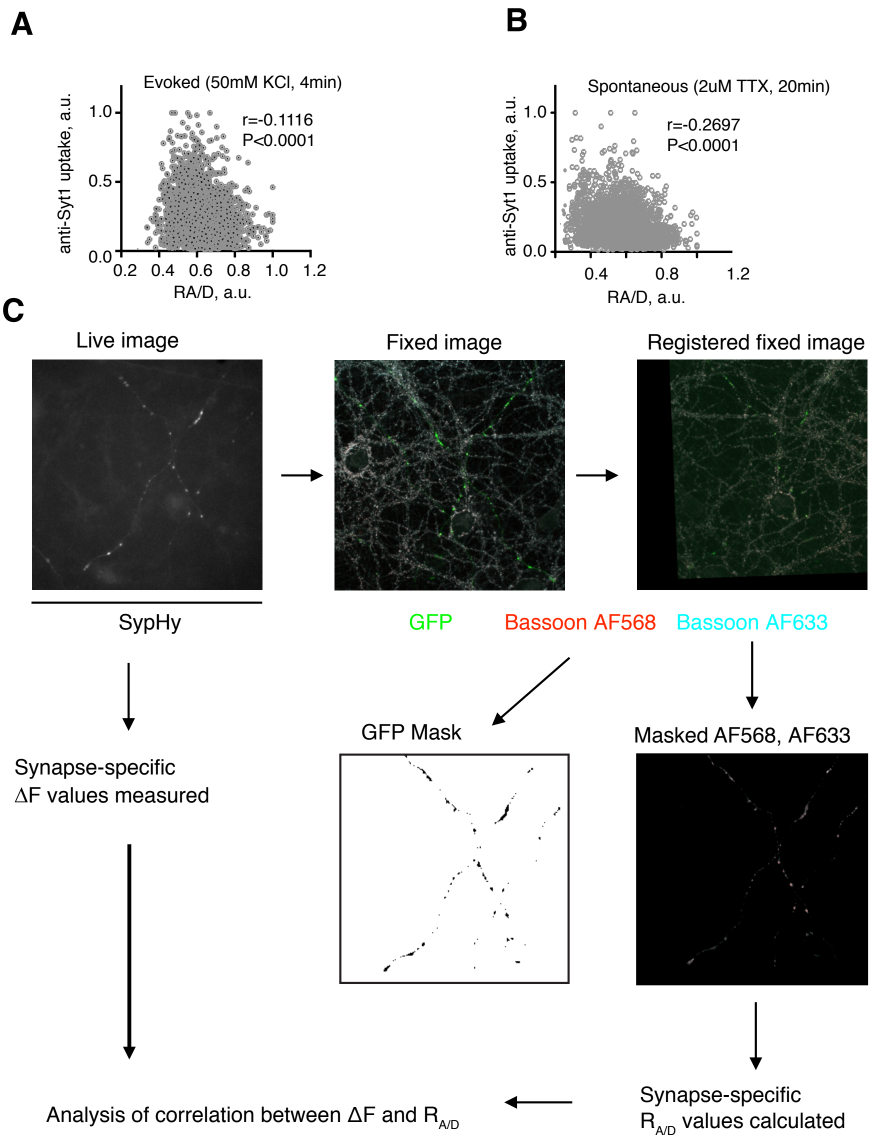

Figure S6. Supporting data for Fig. 5. Correlating AZ matrix clustering with presynaptic function. (**A**), Evoked vesicle cycling negatively correlates with AZ matrix density. Evoked vesicle cycling was induced by depolarizing with 50mM K^+ for 4min. (**B**), Spontaneous vesicle cycling negatively correlates with AZ matrix density. Spontaneous vesicle cycling was isolated by applying 2μM TTX for 20min. (**C**), Schematics of the correlated live-fixed imaging of presynaptic structure and function. Neurons expressing SypHy are imaged live under stimulation by 40APs at 20Hz. From this, ΔF responses of individual synapses are measured. Neurons are then fixed and stained for ratiometric imaging of Bsn (AF568/AF633) and for GFP to amplify the SypHy signal. All fixed image channels undergo affine transformation to align with the live image. The registered fixed GFP image is thresholded to generate a mask, which is applied to registered AF568 and AF633 images, before $R_{A/D}$ values are calculated for each synapse.

Full Methods

Cell culture

Dissociated hippocampal neuronal cultures were prepared from E18 rat embryos, plated onto poly-L-lysine-coated glass coverslips and maintained according to the standard mixed culture protocol. All experiments involving neurons were carried out at 16-21 days in vitro. COS7 cells were also grown according to a standard culture protocol. For confocal imaging, cells were plated onto 13mm round glass coverslips (thickness 1.0) placed in 35mm Petri dishes (4/dish). For STORM imaging, cells were plated onto 18mm round glass coverslips (Carl Zeiss, Germany), thickness 1.5. For live imaging of SypHy cells were plated on 35mm Grid500 μ-Dishes (Ibidi, Germany).

Reagents

Cell culture media was from Invitrogen. Poly-L-lysine was from Sigma. The following primary antibodies were used:

Antigen	Host species	Company	Catalog no.	KO verified? (v for yes)
Bsn	Mouse	Abcam	82958	v
Bsn	Rabbit	Cell Signaling	6897	
Bsn	Mouse	Synaptic Systems	141021	
Cav2.1	Rabbit	Synaptic Systems	152103	v
Cav2.2	Rabbit	Alomone	ACC-002	
Cav2.3	Rabbit	Alomone	ACC-006	
Pclo	Rabbit	Synaptic Systems	142002	v
TfR	Mouse	Abcam	111816	
RIM1/2	Rabbit	Synaptic Systems	140203	
Psd95	Mouse	Abcam	99009	
Geph	Mouse	Synaptic Systems	147021	v
Syt-1	Rabbit	Synaptic Systems	105102	
CASK	Mouse	Abcam	99039	v
GFP	Mouse	Abcam	1218	
GFP	Rabbit	Abcam	290	
GFP	Chicken	Abcam	13970	

Secondary antibodies were from Jackson Immunoresearch (USA). AlexaFluor (AF)568, AF647-conjugated Phalloidin and FM1-43fx were from Molecular Probes. APV, NBQX, TTX were from Tocris (UK). AM251, ACEA, Latrunculin A, Jasplakinolide were from Sigma-Aldrich (UK).

Ratiometric imaging assay for nanoscale clustering

Rationale (see Fig. 2A): A protein of interest (red circles) is labeled with a monoclonal primary antibody (grey), which is then labeled with a mixture of two secondary antibodies respectively conjugated to a FRET donor and acceptor dye. The amount of FRET between the fluorophores is dependent on the average distance between them, which in turn reflects the average distance between the molecules of the protein of interest; namely, a more or less clustered distribution will result in a smaller or larger average distance and therefore more or less FRET as visualized by the acceptor/donor ratio $R_{A/D}$.

Protocol: After treatment, coverslips were fixed with 4%PFA in PBS for 15-20min at room temperature (RT) and permeabilized in 0.2%Triton-X100 in PBS supplemented with 5% horse serum for 10min. Subsequent incubations were carried out in the permeabilization buffer. Coverslips were incubated with an appropriate primary antibody for 60min at RT, washed 4 times in PBS and incubated with a 1:1 mixture of donor- and acceptor-conjugated secondary antibodies at a concentration of 0.3μg/ml each for 60min at RT. AF568 and AF633/AF647/DyLight649 were used as donor and acceptor respectively. Coverslips were then mounted in mounting medium (Southern), allowed to dry for 30min at RT and imaged on a Zeiss LSM710 microscope equipped with a standard set of lasers through a 63x oil objective. Excitation wavelengths were 488, 543 and 633nm. Bandpass filters were set at 500-550 (AF488), 560-615 (AF568) and 650-720nm (AF633, AF647, DyLight649). Image acquisition was typically carried out at the 12-bit rate. Settings were optimized to ensure appropriate dynamic range, low background and sufficient signal/noise ratio. To quantify $R_{A/D}$, 20μm-long regions of interest (ROI) were selected alongside proximal dendrites of neurons manifesting pyramidal morphology, and the ratio between acceptor and donor intensity was quantified after appropriate background subtraction. 3-5 ROI/image were chosen for analysis. To quantify synapse-specific $R_{A/D}$, puncta of fluorescence were individually processed in the same manner.

Sensitized emission FRET imaging

This was carried out essentially as described before (Glebov and Nichols, 2004), with AF568 and AF633 as a donor-acceptor pair. Briefly, FRET values in stretches of dendrites were calculated in samples labeled with both fluorophores, corrected for spectral bleedthrough, and divided by intensity in either donor or acceptor channel to yield FRET ratios.

Optogenetic stimulation

Primary neuronal cultures were sparsely transfected with ChR2-YFP, a gift from K. Deisseroth (http://www.optogenetics. org), at 7DIV using Effectene (QIAGEN, UK). At 15-17DIV cultures were prepared 2h before photostimulation by supplementing the culture medium with additional antioxidants (3.2μM glutathione (Fisher Scientific, UK), 77nM superoxide dismutase, 10nM catalase, 100μM Trolox, 110μM Vitamin C (all from Sigma

Aldrich, UK)). 12-well culture plates were placed directly on top of collimator-topped blue LEDs (Royal Blue Luxeon K2, Philips LumiLEDs; 455 ± 10 nm; ~1 mW mm^{-2} at the coverslip surface), powered by a DC/DC LED driver (Recom) and controlled by a digital I/O device (USB-6501, National Instruments) and custom-written software. Photostimulation was carried out for 48 hours using two different paradigms: a '1Hz burst' stimulation pattern consisting of 5 flashes at 20Hz delivered every 5s or '1Hz sparse' stimulation pattern consisting of 1 flash every second. In both cases, flashes were delivered at 40% LED intensity for 20ms, ensuring that at least one spike occurred per flash. Control plates were loosely covered in tin foil to avoid stimulation from neighboring culture dishes and placed on non-functional LEDs.

Quantification of synapse-specific correlation

Coverslips were processed as for ratiometric imaging. AF405-conjugated anti-rabbit secondary antibody was used to visualize RIM, CASK and $Ca_V2.1$. To maximize the dynamic range, image acquisition was carried out at the 16-bit rate. To reveal AZ morphology, images were thresholded in ImageJ using the "Moments" algorithm. Individual AZs were selected using the "Analyze particles" plugin. To exclude the overlapping AZs from the sample, the particle size selection criteria were set to 0.1-2μm^2. Intensities in all three channels were then quantified in a particle-specific manner, yielding synapse-specific values for the level of the protein of interest as well as the donor and acceptor intensities for Bsn. After appropriate background subtraction, $R_{A/D}$ was quantified for each synapse and RIM (or CASK, or $Ca_V2.1$) intensity was plotted against $R_{A/D}$. Before pooling, the data was normalized to maximal values in fixed-cell imaging experiments or median value in live-fixed correlative imaging experiments.

Anti-Synaptotagmin-1 antibody uptake assay

Antibody uptake assay was performed essentially as described before. Neurons were labeled with 1/100 mouse monoclonal antibody (isotype IgG3) against the luminal/extracellular domain of Synaptotagmin-1 (Syt-1) in culture medium at 37°C. To label the spontaneously cycling synaptic pool, labeling was carried out for 20' in the presence of 2μM TTX. Coverslips were acid-washed to remove the surface label, fixed, permeabilized and stained for Syt-1 and $R_{A/D}$; Syt-1 and Bsn signals were visualized using the appropriate isotype-specific secondary antibodies (AF488-anti-IgG3, AF568-anti-IgG2a and AF647-anti-IgG2a respectively).

Live imaging of presynaptic vesicle cycling

Neurons were sparsely transfected at 7DIV with CMV::SypHy (a gift from Leon Lagnado (Addgene plasmid # 24478)) for imaging presynaptic vesicle release using Effectene (QIAGEN, UK), and imaged at 17-21DIV. For imaging and stimulation the neuronal growth medium was replaced with HEPES buffered saline (HBS; 139mM NaCl, 25mM KCl, 10mM HEPES, 10mM D-Glucose, 2mM $CaCl_2$, 1.3mM $MgCl_2$; pH7.3, 290 mOsm) with 2,3-Dioxo-6-nitro-1,2,3,4-tetrahydrobenzo[*f*]quinoxaline-7-sulfonamide (NBQX), 0.025mM amino-5-phosphonovaleric acid (APV) and 6-Imino-3-(4-methoxyphenyl)-1(6*H*)-pyridazinebutanoic acid hydrobromide (Gabazine) (all Tocris, UK). A field stimulation insert with platinum electrodes (RC-37WS, Warner Instruments, USA) was placed inside the dish. Neurons

were imaged on an inverted Olympus IX71 microscope, equipped with a 60x/1.42 NA oil objective. SypHy was excited using a 470nm wavelength LED (CoolLED, UK) and imaged using the appropriate filters (Excitation: 470+/-20nm; Dichroic: 495 nm long-pass; Emission: 525+/-25nm; Chroma Filters, USA). Time-lapse images were acquired at the frequency of approximately 13Hz using an Evolve 512 EMCCD camera (Photometrics, USA) controlled by Slidebook software (Intelligent Imaging Innovations, USA). Slidebook was also used to trigger a frame-locked delivery of a 1ms, 80V pulse 40 times at 50ms intervals (*i.e.* 20Hz) for SypHy, where each pulse approximates a single action potential (AP)(Zhao et al., 2011). Several regions in each dish were imaged, within a maximum imaging period of 1 hour.

Images were analyzed using custom written Matlab codes (Mathworks), in which background-subtracted fluorescence intensity values were calculated from the mean of a square 8x8 pixel ROI selected for each presynaptic bouton identified by SypHy fluorescence. ΔF values were calculated as the change in signal intensity from the mean of the baseline (all frames preceding the stimulus), and the peak response was defined as the maximum ΔF within 70 frames of the stimulus. Only synapses in which the peak ΔF response was greater than 3 standard deviations of the baseline were analyzed. ΔF values were normalized to the mean peak response of all synapses in each image before pooling.

Live imaging of presynaptic calcium influx and agatoxin blockade

A synapsin::SyGCaMP6F plasmid was constructed by replacing GCaMP3 in SyGCaMP3 (Nikolaou et al., 2012) with GCaMP6F (a gift from Douglas Kim, (Addgene plasmid #40755)) and replacing the promoter. Transfection with this construct and imaging of presynaptic calcium influx were carried out as for imaging of vesicle cycling using SypHy, with the exception that the stimulation applied was 10APs at 20Hz, repeated three times one minute apart. After this, half of the imaging media was removed and replaced with the same volume of media containing 200nM ω-agatoxin IVA (Alomone, Israel) and mixed by pipetting up and down, yielding a final concentration of 100nM. After 5 minutes of incubation in agatoxin the three 10AP 20Hz stimuli were repeated. Analysis was also carried out as above. Responses were averaged across the three trials and ΔF values were normalized to the baseline fluorescence of each ROI to give ΔF/F values.

Correlative live-fixed imaging of presynaptic structure and function

The schematic of the approach is presented in Figure S6. After live imaging of SypHy responses, cells were fixed and stained for ratiometric imaging of Bsn as above, with the addition of chicken anti-GFP primary antibody (Abcam, UK) and anti-chicken AF488 conjugated secondary antibody to amplify the SypHy signal. Dishes were mounted with Ibidi mounting medium (Ibidi, Germany). Regions that had previously been imaged live were relocated on an inverted Nikon Eclipse Ti spectral confocal microscope equipped with a 60x/1.40 NA oil objective and NIS Elements software. Excitation wavelengths were 488, 561 and 636nm. Emission bandpass filters were set at 500-550nm (AF488), 570-620nm (AF568) and 662-737nm (AF633). Optical sections were taken at

0.1μm steps to create z-stacks, from which maximum projections were generated in ImageJ. To calculate $R_{A/D}$ values for all synapses in the image, thresholding and particle analysis was carried out as for synapse-specific quantification. From this, the median $R_{A/D}$ value of each image was calculated for later use in normalization. Fixed images were also analyzed using custom written Matlab codes (Mathworks). Corresponding live SypHy images and fixed GFP images were first registered by manual selection of landmarks in both images followed by affine transformation of the fixed image. ROIs selected on live SypHy images were then scaled and overlaid on the registered fixed GFP image for manual confirmation of the position. The registered GFP image was then thresholded using the 'Moments' algorithm in ImageJ to generate a mask, which was applied to registered AF568 and AF633 images to ensure that neighboring synapses were not included when ROIs were applied to these channels. Any ROIs falling outside of the GFP positive mask were also excluded. Intensity and $R_{A/D}$ values for each ROI were calculated from the masked images. $R_{A/D}$ values were normalized to the mean $R_{A/D}$ of all synapses in the image before pooling.

Single color STORM imaging

Samples were processed in the same manner as for ratiometric imaging, except that the only secondary antibody used was the AF647-conjugated anti-mouse at 1μg/ml. Samples were then incubated in the STORM imaging buffer with MEA (for recipe see http://www.nikoninstruments.com/en_GB/Products/Microscope-Systems/Inverted-Microscopes/N-STORM-Super-Resolution/(brochure)) in the following manner. The 22mm square coverslips were lifted from their dish, excess buffer was removed by blotting with paper and the coverslips were upturned onto 30μl of the imaging buffer placed on a glass slide. Coverslips were secured on the slide using Vaseline, and excess imaging buffer was blotted out with paper. With glass-bottomed dishes, 300μl imaging buffer was added directly onto the dishes. In both cases, imaging was performed immediately after mounting. Imaging was performed on an N-STORM Nikon Ti-E TIRF inverted microscope equipped with an Andor iXon DU897 EMCCD camera and a Perfect Focus system, running on the NIS Elements software. Imaging was carried out at the 12-bit rate through a 100x oil objective with a 647nm laser. The ROI was set at 128x128 pixels, with the pixel size of 160nm. The ROI was pre-bleached at 100% laser power until individual blinking events could be clearly distinguished, and subsequently imaged in the near total internal reflection fluorescence (TIRF) mode for 2000-5000 frames at a rate of 50frames/s. The resulting data was exported into the TIFF format. The positions of individual molecules were determined using the QuickPALM plugin (Henriques et al., 2010) with the default settings on the ImageJ platform.

Dual color STORM imaging

Dual color imaging was performed on a custom-built setup as described before (Winterflood et al., 2015). In brief, a 473 nm laser (100 mW, Laserglow Technologies) was used for activation and a 643 nm laser (150 mW, Toptica Photonics) for imaging. Emission light was filtered by two bandpass emission filters (700/75 nm, Chroma) and a longpass dichroic beam splitter (690 nm, AHF Analysetechnik) was used to split the emission light. The imaging buffer consisted of 0.1 M MEA/0.2 M Tris, pH 8.0 with 5 % (w/v) glucose, 0.25 mg/ml glucose-oxidase and 20 μg/ml catalase. Imaging was performed in objective-type near-TIRF mode. A minimum of 20000 frames with an exposure time of 20-33ms was

recorded. An image-correlation based drift-correction was employed. All data analysis was performed in MATLAB (Mathworks). To differentiate between AF647 and CF680 single molecule fluorescence profiles, the normalized intensity ratio was calculated for all localization pairs for the color-assignment using $r=(I_l-I_s)/(I_l+I_s)$, where I_l and I_s are the fluorescence intensities determined by maximum-likelihood fitting for the long and short wavelength channels respectively. The cut-off criteria (green and red lines in Fig. S1C) were then selected manually for each image to minimize the conflation of the fluorophores' identities. NNLEs were measured using a *knnsearch* function in Matlab. Data was then processed using the off-gap method to remove spurious localizations resulting from photoblinking or fluorophore reactivation (Annibale et al., 2011; Williamson et al., 2011). Areas that unambiguously conformed to the previously reported morphological criteria for AZ in a "face-on" (*i.e.* flat) configuration (Dani et al., 2010; Schikorski and Stevens, 1997) were manually selected for further analysis. Each experiment was repeated thrice.

Correlation analysis of spatial distribution in STORM data

Selected AZ (15/condition, 3 experiments) were processed in Matlab into three bins at 20, 50 and 100nm/pixel respectively, resulting images were exported in TIFF format and further processed in ImageJ using the Colocalization Test plugin. To determine the non-randomness of the distribution, the observed correlation coefficient was then compared using the Costes method (Costes et al., 2004) to the mean value from 100 randomized iterations. The outcomes were then classed into three categories: if the observed correlation coefficient value was significantly lower or higher than the randomized mean, the distributions were considered non-randomly exclusive or inclusive respectively, while the value not significantly different from the randomized mean was considered indicative of a random distribution. Fisher's exact test was subsequently used to assess the statistical significance of the differences in proportions of AZ belonging to these categories.

Quantitative clustering analysis

The list of x and y coordinates of individual molecules was analyzed for clustering using Ripley's K-function and Getis and Franklin's local point pattern analysis. For this process, 3x3um representative square regions were chosen for analysis. In Ripley's K-function, concentric circles are drawn around each point and the number of other points encircled is then counted. This value is then normalized based on the total molecular density such that the K-function at a circle radius r is calculated as:

$$K(r) = A\sum_{i=1}^{n}\sum_{j=1}^{n}\frac{\delta_{ij}}{n^2}\ where\ \delta_{ij=}\begin{Bmatrix}1\ if\ d_{ij} < r\\ 0\ else\end{Bmatrix}$$

Where A is the analyzed area, n is the total number of points within that ROI and d_{ij} is the distance between two points i and j. The K-function is then linearized such that it scales with circle radius rather than area, giving the L-function:

$$L(r) = \sqrt{\frac{K(r)}{\pi}}$$

A completely spatially random (CSR) distribution of points will have L(r)=r at all r. We therefore subtract r and plot L(r)-r versus r. In this case a CSR distribution is a straight line at L(r)-r=0. Positive values of L(r)-r indicate the spatial scale of clustering: the greater the value of L(r)-r the more clustered the distribution.

In order to generate the cluster heat maps and extract cluster parameters, Getis and Franklin's local point pattern analysis was used. Here, the value of L(r) is calculated for each point in the ROI individually for a single specified spatial scale (circle radius). A scale of 50 nm was selected. The equation therefore becomes:

$$L(50) = \sqrt{A\sum_{i=1}^{n}\left(\frac{\delta_{ij}}{n}\right)/\pi} \quad where\ \delta_{ij} = \begin{Bmatrix} 1\ if\ d_{ij} < 50 \\ else\ 0 \end{Bmatrix}$$

Values of L(r) at each molecule location were then interpolated onto a 5nm resolution grid to generate the cluster maps. To extract cluster parameters, points and areas of this map were designated as being within a cluster if the value of L(r) is above a binary threshold (L(r)>XX). This method has previously been demonstrated for the analysis of clustering in single-molecule dSTORM data sets (Williamson et al., 2011).

Statistics

Statistical analysis was carried out using GraphPad Prism5.0. Sample distribution was assessed using D'Agostino and Pearson's omnibus normality test; to assess the significance of differences between datasets, Mann-Whitney test was used unless noted otherwise. Error bars indicate 10-90 percentile range unless noted otherwise. ***P<0.001, **P<0.01, *<P<0.05.

Supplementary References

Annibale, P., Vanni, S., Scarselli, M., Rothlisberger, U., and Radenovic, A. (2011). Identification of clustering artifacts in photoactivated localization microscopy. Nat. Methods *8*, 527–528.

Costes, S. V, Daelemans, D., Cho, E.H., Dobbin, Z., Pavlakis, G., and Lockett, S. (2004). Automatic and quantitative measurement of protein-protein colocalization in live cells. Biophys. J. *86*, 3993–4003.

Dani, A., Huang, B., Bergan, J., Dulac, C., and Zhuang, X. (2010). Superresolution imaging of chemical synapses in the brain. Neuron *68*, 843–856.

Glebov, O.O., and Nichols, B.J. (2004). Lipid raft proteins have a random distribution during localized activation of the T-cell receptor. Nat. Cell Biol. *6*, 238–243.

Henriques, R., Lelek, M., Fornasiero, E.F., Valtorta, F., Zimmer, C., and Mhlanga, M.M. (2010). QuickPALM: 3D real-time photoactivation nanoscopy image processing in ImageJ. Nat. Methods *7*, 339–340.

Nikolaou, N., Lowe, A.S., Walker, A.S., Abbas, F., Hunter, P.R., Thompson, I.D., and Meyer, M.P. (2012). Parametric functional maps of visual inputs to the tectum. Neuron *76*, 317–324.
